# Supplementary material for: Combining Mg–Zn–Ca Bulk Metallic Glass with a Mesoporous Silica Nanocomposite for Bone Tissue Engineering
Source: Pharmaceutics. 2022 May 17;14(5):1078. doi: 10.3390/pharmaceutics14051078 (PMC9145403; doi:10.3390/pharmaceutics14051078)
Supplement: Supplementary file 1 [file pharmaceutics-14-01078-s001.zip › pharmaceutics-1717093-supplementary.pdf]

Supplementary Materials

Combining Mg-Zn-Ca Bulk Metallic Glass with a Mesoporous Silica Nanocomposite for Bone Tissue Engineering

Yun Shin Chu, Pei-Chun Wong, Jason Shian-Ching Jang, Chih-Hwa Chen, Si-Han Wu *

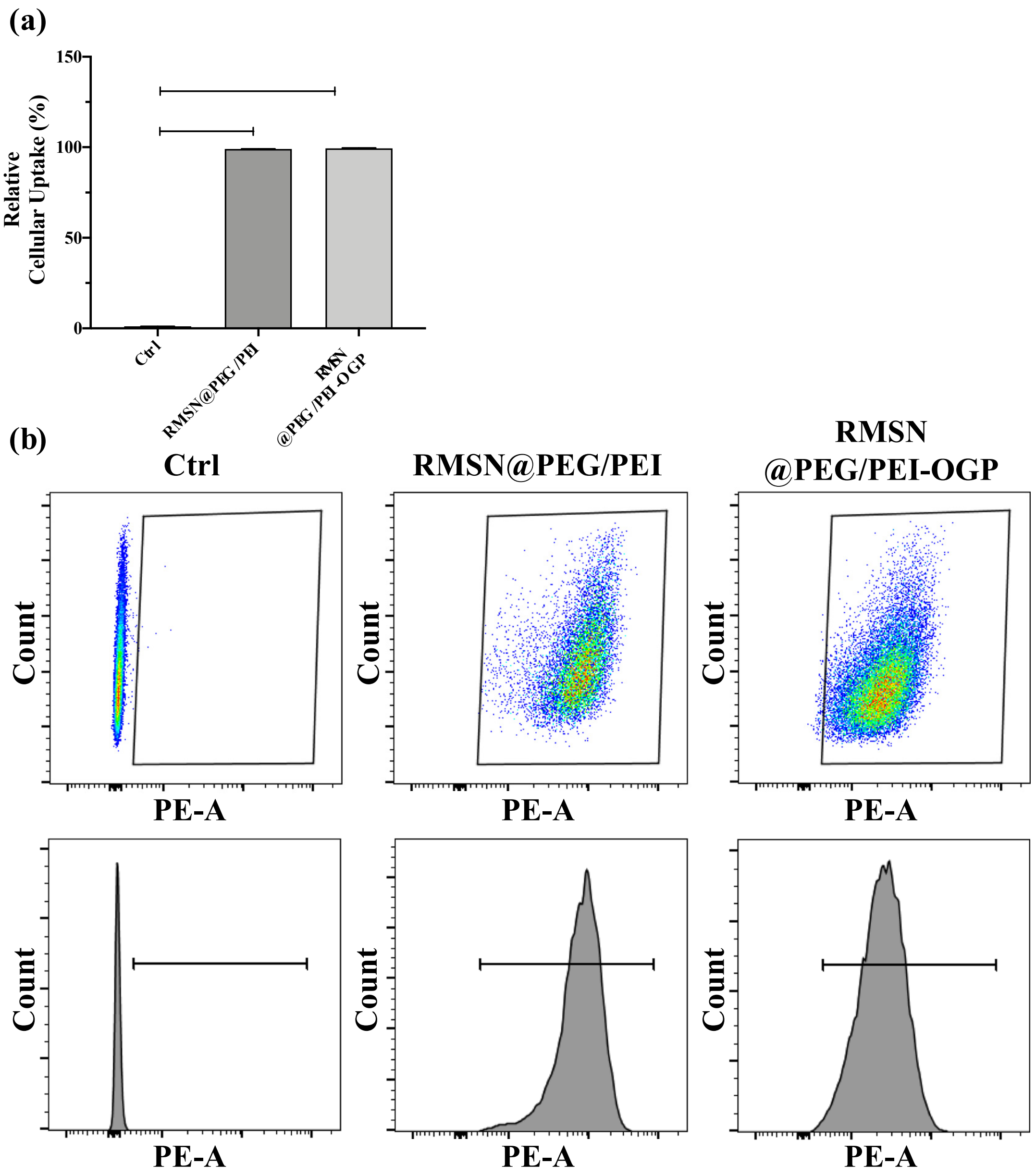

Supplementary Figure S1. (A) Flow cytometric analysis and (B) the quantitative chart of flow cytometry analysis of MC3T3-E1 cells at 24 h post-treatment with nanoparticles. The amount of nanoparticles were 50 μg/mL. (n=3 per group) [Data are mean ± S.D., **** $p < 0.0001$].

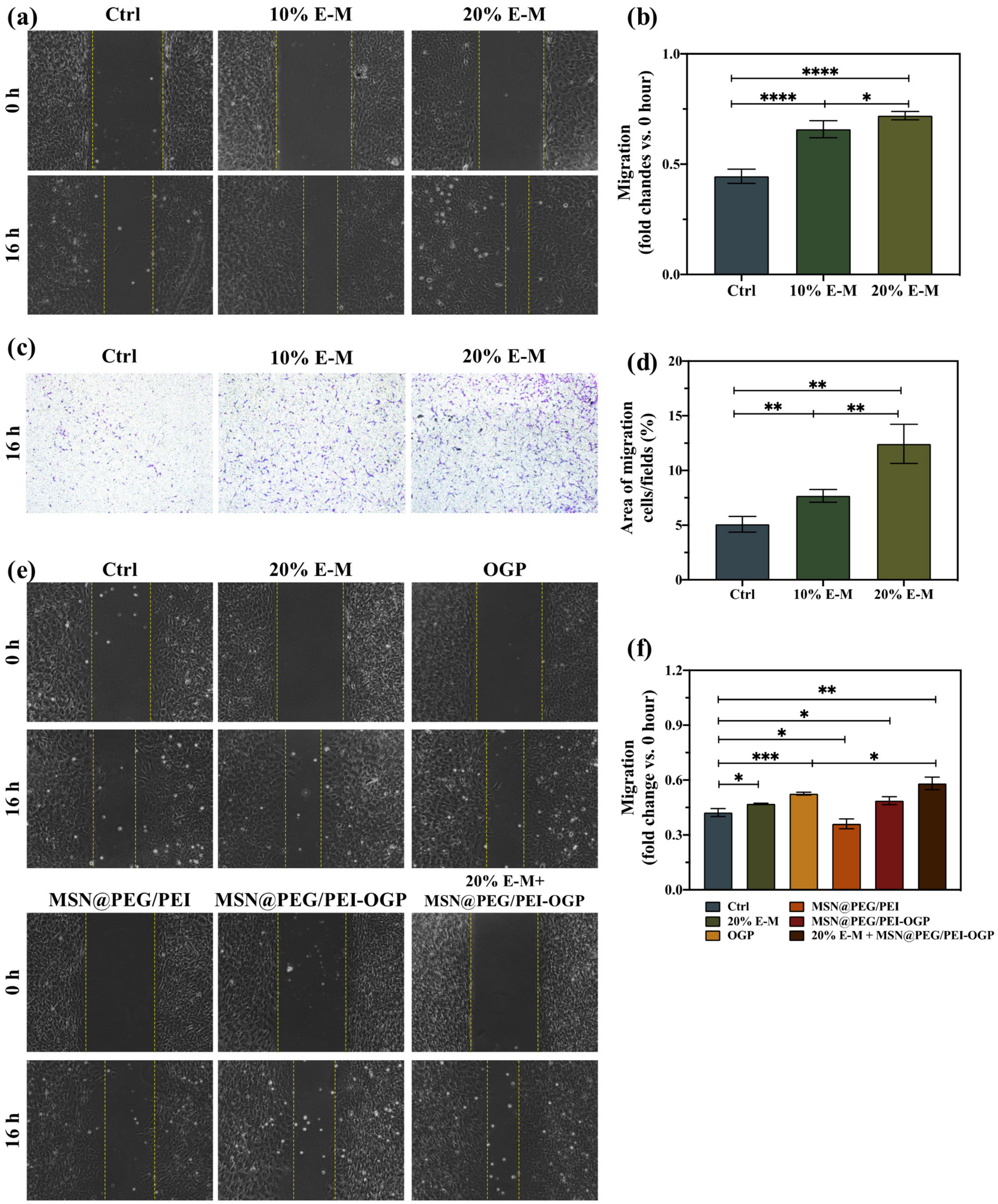

Supplementary Figure S2. (A) The morphology of MC3T3-E1 cells in wound healing assay with different ratio of extracted-αMEM. (B) The transwell assay of different concentration of extracted-αMEM. (C) The morphology of MC3T3-E1 cells at 24 h post-treatment with different condition of wound healing assay. The quantitative chart of (D) wound healing assay and (E) transwell assay with different ratio of extracted-αMEM. (F) The quantitative chart of post-treatment with different condition

of wound healing assay. The amount of nanoparticles were 100 µg/mL. (n=3 per group). [Data are mean ± S.D., * $p < 0.05$, ** $p < 0.01$, *** $p < 0.001$, **** $p < 0.0001$].

Supplementary Table S1. DLS measurements of MSNs. (n=3 per group). [Data are mean ± S.D.].

**Sample**	**Z-average (d.nm) / PDI**	
	**D_h in PBS**	**D_h in Water**
**MSN@PEG/PEI**	91.66 ± 0.707/ 0.094	83.62 ± 1.511/ 0.104
**MSN@PEG/PEI-OGP**	107.8 ± 1.054 / 0.124	100.3 ± 1.059 / 0.100

Supplementary Table S2. Complete blood count of the rabbits implanted treated with different condition after 4 weeks post-surgery. (n=3 per group). [Data are mean ± S.D.].

**Item**	**References**	**$Mg_{66}Zn_{29}Ca_5$ BMG**	**$Mg_{66}Zn_{29}Ca_5$ BMG + MSN@PEG/PEI-OGP**	**$Mg_{66}Zn_{29}Ca_5$ BMG + OGP**
**Lymphocytes (LYM)**	**35~85% ($3 – 9 \times 10^9$ /L)**	36.57 ± 17.39 (2.53 ± 0.82)	51.43 ± 12.69 (3.00 ± 1.06)	65.77 ± 3.59 (4.94 ± 0.64)
**White Blood Cells (WBC)**	**$5\text{-}13 \times 10^9$ /L**	7.73 ± 3.18	5.82 ± 1.21	7.55 ± 1.21
**Neutrophils (NEU)**	**20-75%**	47.63 ± 17.18	32.80 ± 8.03	23.30 ± 3.05
**Monocyte (Mono)**	**1-4% ($< 0.5 \times 10^9$ /L)**	6.93 ± 0.58 (0.53 ± 0.2)	8.77 ± 3.48 (0.52 ± 0.27)	5.17 ± 0.74 (0.39 ± 0.10)
**Eosinophils (EO)**	**0-4%**	0.47 ± 0.55	0.93 ± 0.38	1.77 ± 0.15
**Basophils (BASO)**	**2-7%**	8.40 ± 1.90	6.07 ± 1.67	4.00 ± 0.62
**Red Blood Cells (RBC)**	**$4\text{-}7 \times 10^{12}$ /L**	5.13 ± 0.86	4.93 ± 0.45	6.26 ± 0.44
**Hemoglobin (HB)**	**10.0-15.5 g/dl**	9.67 ± 1.69	10.60 ± 1.04	13.20 ± 0.98
**Mean Corpuscular Volume (MCV)**	**50~75 mm^3**	58.33 ± 0.81	67.03 ± 1.50	66.47 ± 1.54
**Platelets (PLT) ×**	**$250\text{-}270 \times 10^3$ /mm^3**	308.67 ± 135.49	244.33 ± 57.07	223.67 ± 53.98

Supplementary Table S3. Serum biochemistry of the rabbits treated with different condition after 4 weeks post-surgery. (n=3 per group). [Data are mean ± S.D.].

**Item**	**References**	**$Mg_{66}Zn_{29}Ca_5$ BMG**	**$Mg_{66}Zn_{29}Ca_5$ BMG + MSN@PEG/PEI-OGP**	**$Mg_{66}Zn_{29}Ca_5$ BMG + OGP**
**Creatinine (CREA)**	**0.8-1.8 mg/dL**	0.73 ± 0.35	0.87 ± 0.12	1.13 ± 0.06
**Blood Urea Nitrogen (BUN)**	**10-24 mg/dL**	18.00 ± 8.19	17.00 ± 6.24	17.67 ± 4.16
**Total Bilirubin (TBIL)**	**0.3-0.8 mg/dL**	0.27 ± 0.21	0.23 ± 0.15	0.17 ± 0.12
**Albumin (ALB)**	**2.7-4.6 g/dL**	2.57 ± 0.32	3.00 ± 0.30	3.03 ± 0.15
**Alanine Aminotransferase (ALT)**	**31-53 U/L**	61.33 ± 23.18	54.00 ± 7.55	44.00 ± 11.27
**Alkaline Phosphatase (ALKP)**	**70-145 U/L**	39.67 ± 16.29	44.76 ± 4.73	58.00 ± 10.15
**Magnesium (Mg)**	**1.95-2.9 mg/dL**	3.29 ± 0.73	2.28 ± 0.46	2.56 ± 0.03
